# Regulatory Status of Cannabidiol in the United States: A Perspective

**DOI:** 10.1089/can.2018.0030

**Published:** 2018-09-27

**Authors:** Jamie Corroon, Rod Kight

**Affiliations:** ^1^ The Center for Medical Cannabis Education, Del Mar, California.; ^2^ Helfgott Research Institute, National University of Natural Medicine (NUNM), Portland, Oregon.; ^3^ Kight Law Office LLC dba Kight On Cannabis, Asheville, North Carolina.

**Keywords:** cannabidiol, CBD, hemp, marijuana, *Cannabis*, phytocannabinoids

## Abstract

Cannabidiol (CBD) is 1 of > 100 cannabinoids found in *Cannabis sativa* L. (*Cannabis spp.* or *Cannabis*). Despite its complex and rapidly evolving regulatory status in the United States, projected retail sales of CBD products—hemp, cannabis and pharmaceutical—are as high as $1.9 billion by 2020. CBD products can currently be purchased online, over the counter, and at cannabis-specific dispensaries throughout most parts of the country, despite the fact that CBD is presently deemed a Schedule I controlled substance by the U.S. Drug Enforcement Administration and renounced as a dietary supplement ingredient by the U.S. Food and Drug Administration (FDA). These products are largely unregulated, and are being used predominantly to treat specific medical conditions. Recent FDA approval of Epidiolex (CBD) as a treatment for certain pediatric seizure disorders will prompt scheduling of CBD and likely alter FDA enforcement of the Federal Food, Drug, and Cosmetic Act (FD&C), which to date has mostly been in the form of warning letters. Persuasive legal arguments contend that CBD's legal status is based on its source. According to these arguments, there are three legal sources. CBD-derived from: (1) parts of the *Cannabis* plant that do not meet the definition of cannabis in the Controlled Substances Act (CSA); (2) imported “non-psychoactive hemp”; and (3) “Industrial hemp” cultivated as part of a state pilot program per the 2014 Farm Act. Although CBD's lawful status with respect to the CSA appears to be expanding, its future regulatory status with respect to the FD&C Act is difficult to predict.

On June 25, 2018, the U.S. Food and Drug Administration (FDA) announced its first-ever approval of a *Cannabis*-derived pharmaceutical drug. Epidiolex (cannabidiol or CBD), an oral solution, was approved for the treatment of two rare and severe seizure disorders, Lennox–Gastaut syndrome and Dravet syndrome.^1^ Availability of Epidiolex is pending Drug Enforcement Administration (DEA) scheduling of CBD, which is expected before September 23, 2018. This historic decision may have important consequences for multiple constituencies, including patients, consumers of over-the-counter (OTC) CBD products, healthcare providers, clinical researchers, industry stakeholders, and governmental agencies, including law enforcement. Understanding the therapeutic potential of CBD has been thwarted to date by inconsistent and conflicting federal and state regulations dating back to the passage of the Controlled Substances Act (CSA) in 1970.

CBD is 1 of >100 cannabinoids found in *Cannabis sativa* L. (*Cannabis* spp. or *Cannabis*), a plant more well-known colloquially as marijuana or hemp, herein referred to as “cannabis.”^2^ CBD was first isolated in 1940 and characterized structurally in 1963.^3,4^ With projected retail sales of CBD products—hemp, cannabis and pharmaceutical—as high as $1.9 billion by 2020, CBD is poised to become the darling of the medical *Cannabis* movement.^5^

Despite its rapidly growing popularity and use, the regulatory status of CBD in the United States is convoluted. The source of CBD is critically important in determining its legal status. The most common source, botanically speaking, is the plant *Cannabis sativa* L. (*Cannabis*), which encompasses both cannabis and hemp. From a regulatory standpoint, the difference between cannabis and hemp is the chemical composition, specifically as it relates to the concentration of delta-9 tetrahydrocannabinol (THC), the primary intoxicating compound found in *Cannabis*. By this classification, hemp is a chemovar of *C. sativa* with low concentrations of THC. Although limitations on THC concentrations for hemp differ internationally, the THC concentration in the United States cannot exceed 3/10ths of 1% (0.3%). Hemp-derived and cannabis-derived CBD each has its own unique regulatory status and consequent legal implications.

As a compound extracted from cannabis, CBD is currently deemed a Schedule I controlled substance by both the FDA and the DEA pursuant to the 1970 CSA, which means that it has no currently accepted medical use, a lack of accepted safety for use under medical supervision, and a high potential for abuse.^1,6–10^ This classification is in stark contrast to the position of the World Health Organization, which issued a preliminary report in November, 2017 describing CBD's efficacy for treating a number of health issues.^11^ Ironically, it also conflicts with the position of U.S. Department of Health and Human Services (HHS), of which the FDA is a subagency. The HHS owns a patent on cannabinoids as antioxidants and neuroprotectants, in which CBD is explicitly named.^12^

CBD can also be extracted from hemp. Historically, hemp has been bred as an industrial crop to produce fabrics, rope, and other textiles from its long stalks.^13^ Technically speaking, and despite not being explicitly mentioned in the CSA, hemp is a controlled substance unless an exemption applies.

The Agricultural Act of 2014, commonly known as the “2014 Farm Act,” is one such exemption.^14^ It carved out “industrial hemp” as the *C. sativa* plant cultivated pursuant to a state's pilot research program with delta-9 THC concentrations that do not exceed 0.3%. Industrial hemp is an exemption from the CSA definition of cannabis (i.e., marijuana) because the 2014 Farm Act expressly preempts the CSA.^15^ CBD derived from industrial hemp is lawful under federal law and the laws of some states.^16^

Despite the distinction carved out by the Farm Act, the legal status of CBD was called into question on December 14, 2016, when the DEA announced “the Establishment of a New Drug Code for Marihuana Extract,” commonly known as the Marijuana Extract Rule (MER).^17^ The MER defined unlawful cannabis extract as “an extract containing one or more cannabinoids that has been derived from any plant of the genus Cannabis…” With respect to CBD, the MER specifically states that CBD “fall[s] within the new drug code.”^17^ This action prompted a national trade organization, the Hemp Industries Association (HIA), and several businesses in the hemp industry, to file a petition^18^ against the DEA to strike the MER or, in the alternative, obtain clarification of it.^15^

In apparent response, the DEA issued a “Clarification of the New Drug Code for Marijuana Extract” (Clarification) on March 14, 2016, which states that, “[t]he new drug code includes only those extracts that fall within the CSA definition of marijuana.”^19^ Further clarification was provided on May 22, 2018, when the DEA issued an internal directive that stated, “Products and materials that are made from the cannabis plant and which fall outside the CSA definition of marijuana (such as sterilized seeds, oil or cake made from the seeds, and mature stalks) are not controlled under the CSA.”^20^ Although these statements clarified that CBD derived from a source other than cannabis was lawful, they did not specifically state that CBD from industrial hemp was lawful. For this reason, the clarification did not resolve the litigation.

In May, 2018, the court issued a ruling denying the plaintiffs' request to strike down the MER on procedural grounds and reiterated that the Farm Act preempts contrary provisions in the CSA.^21^ This ruling maintained the status quo.^22^

Currently, domestically cultivated hemp is only federally lawful when cultivated under such a pilot program. In 2017, 23,343 acres of hemp were cultivated across 19 states. As of this writing, 41 states have passed legislation to allow them to take advantage of hemp pilot programs under the 2014 Farm Bill.^23^ Many of these programs authorize commercial sales and marketing of resulting hemp-derived CBD products by private actors licensed to do so.

Hemp could be cultivated lawfully in all states, independent of a pilot program, if The Hemp Farming Act of 2018, S. 2667 (2018 Hemp Bill), passes the U.S. Congress, as it is expected to do this year. The 2018 Hemp Bill has bipartisan support. It was introduced by Senate Majority Leader Mitch McConnell (R-KY) on April 12, 2018, and passed the Senate intact on June 28, 2018, as part of The Agricultural Improvement Act of 2018 (2018 Farm Bill).^24^ Importantly, the 2018 Hemp Bill explicitly includes “cannabinoids” in the definition of lawful hemp.^24^ If passed, it will clarify much of the current confusion surrounding CBD's legal status.

In addition to industrial hemp and *Cannabis* stalks, CBD is also lawful if it is derived from “non-psychoactive hemp” imported into the United States. Nonpsychoactive hemp is the term used by the Ninth Circuit Court of Appeals in a pair of companion cases filed against the DEA by the HIA regarding another DEA rule that would have made it illegal to import any hemp products that contained any THC, including trace amounts.^25,26^

In a February 6, 2004, ruling, the court found that the DEA had exceeded its authority in enacting the rule and struck it down as void and unenforceable.^27^ In its ruling, the court used the term “non-psychoactive hemp,” and in a footnote stated, “The non-psychoactive hemp used in Appellants' products is derived from industrial hemp plants grown in Canada and in Europe, *the flowers of which contain only a trace amount of the THC contained in marijuana varieties grown for psychoactive use*” (*emphasis* added). Confusion remains as to whether the court, in effect, legalized the whole hemp plant for importation, including the “flowering tops,” so long as it contains no more than trace amounts of THC, or whether it simply reiterated the mature stalks exception in a different context. That distinction has never been addressed. Cases addressing hemp, which both preceded and succeeded this ruling, do not resolve the issue.^8^

Despite the confusion and the stance of the FDA and DEA, hemp-derived CBD products can currently be purchased as labeled dietary supplements, both online and OTC, throughout most of the country. In contrast, cannabis-derived CBD products can only be purchased by qualifying patients in states with medical cannabis laws (31 states, and the District of Columbia as of this writing) or by customers in states with recreational cannabis laws (9 and the District of Columbia as of this writing).^28^

To complicate matters further, before the approval of Epidiolex (CBD), the FDA explicitly stated that “CBD products are excluded from the dietary supplement definition” because of CBD's status as an investigational new drug (IND) under the Federal Food, Drug, and Cosmetic Act (FD&C Act).^29^ According to the FDA, the submission of the IND application for Epidiolex by Greenwich Biosciences, the U.S. subsidiary of London-based GW Pharmaceuticals, preceded the sales and marketing of CBD as a dietary supplement.^29^ As a result, CBD cannot be included in a dietary supplement.

This preclusion is not entirely novel. In a somewhat similar case, Biostratum, a pharmaceutical company, requested the FDA to take action against manufacturers of pyridoxamine-containing dietary supplements because Biostratum had submitted an IND application for pyridoxamine dihydrochloride.^30^ It took the FDA 3.5 years to formally conclude that these products were in violation of its regulations. Products containing pyridoxamine and being sold as dietary supplements are not currently permitted.

There is another precedent that informs predictions of how the FDA might approach the sales and marketing of hemp-derived CBD products in the post-Epidiolex era. In April 1997, Pharmanex, a dietary supplement manufacturer, was advised by the FDA that its mevinolin-containing dietary supplement, named Cholestin, was a drug, not a dietary supplement. Mevinolin, also known as monocalin K, is a constituent of red yeast rice and has been shown to lower elevated cholesterol levels.^31^ Mevinolin is chemically identical to lovastatin (brand name Mevacor), an FDA-approved drug manufactured by Merck. The FDA concluded that Cholestin was manufactured to contain concentrations of lovastatin that exceeded traditional red yeast rice products, and the product was thus more similar to a drug than any red yeast rice product available OTC.^32^ Although Cholestin is no longer available, the FDA authorizes the sale of red rice yeast products with naturally occurring concentrations of lovastatin. Many red yeast rice products remain on the market.

In the Cholestin case, the FDA's argument hinged on the concentration of lovastatin in red yeast rice products exceeding some traditional standard. The vast majority of hemp-derived CBD oil products available today contain concentrations by weight of CBD below 5%, as compared to Epidiolex, which is ≥99% CBD.^33^ Given this precedent, it is possible, and even likely, that the FDA will restrict products that are enriched with CBD but not products that contain naturally occurring concentrations of CBD.

With FDA approval of Epidiolex, and the FDA's public proclamation that CBD products are excluded from the statutory definition of a dietary ingredient, the future of online and OTC CBD products is uncertain. The FDA has the authority to enforce the FD&C Act against products that are enriched with CBD. It is worth noting that the FDA is a public health agency with a myriad of competing priorities and a limited enforcement budget. When considering an enforcement action, the FDA weighs multiple factors, including benefits and harms.^29^ To date, harms associated with hemp-derived CBD products have been largely undocumented. It is plausible that the FDA will choose not to exercise its enforcement options. If the FDA does choose to take action however, it would likely reduce consumer access to CBD products. As yet, there has been little meaningful effort on the part of the FDA, or the DEA, to interfere with the widespread OTC sales of CBD products.

In the absence of strict FDA enforcement and oversight, widespread mislabeling of CBD products exists. Independent research has confirmed that the CBD content in almost 70% of CBD products available online could be mislabeled (43% of products were underlabeled and 26% were overlabeled for actual CBD content).^34^ The FDA sent warning letters to 25 companies in 2015–2016 for violations of FDA rule.^35^ The FDA has also sent cease and desist letters to companies for making drug claims about CBD products, including claims that they treat, or even cure, cancer.^36^

## Conclusion

The current legal and regulatory status of CBD is both complex and evolving, particularly with regard to its legal status vis-a-vis the CSA and its regulatory status under the FD&C Act. Myriad factors contribute to this complexity, including convoluted and conflicting regulations at both the federal and state levels, court rulings that have failed to achieve resolution, confusion relating to the definitions of cannabis and hemp, pending legislation, and the FDA's position on CBD as a drug and not a dietary supplement ingredient.

Although the implications of these complexities are widespread, restrictions in clinical research, in particular, have hindered the understanding of important safety and efficacy considerations. As a result, individuals are currently using CBD products to treat medical conditions without the support of informed healthcare providers.^37^

Although the approval of Epidiolex will precipitate scheduling of CBD, it is less clear how it will influence the FDA's enforcement priorities relating to hemp-derived CBD products. Increased enforcement could result in decreased access. In contrast, passage of the 2018 Hemp Bill, which expressly legalizes hemp-derived cannabinoids, including CBD, may prevent enforcement by creating a de facto legal market for hemp-derived CBD products that is separate and distinct from the medical market for Epidiolex. Or, perhaps products with low concentrations of CBD will remain below FDA enforcement priorities, as in the case of red yeast rice.

CBD is currently lawful under certain conditions if derived from a lawful source.^16^ Although its lawful status with respect to the CSA appears to be expanding, its future regulatory status with respect to the FD&C Act is difficult to predict.

## Abbreviations Used

CBD: cannabidiol
CSA: Controlled Substances Act
DEA: Drug Enforcement Administration
FDA: U.S. Food and Drug Administration
FD&C Act: Federal Food, Drug, and Cosmetic Act
HHS: Health and Human Services
HIA: Hemp Industries Association
IND: investigational new drug
MER: Marijuana Extract Rule
OTC: over-the-counter
THC: tetrahydrocannabinol
